# Progress in Medicine

**Published:** 1895-10-12

**Authors:** 


### Progress in Medicine.

### DISEASES OF THE STOMACH.

(*Concluded from Vol. XVIII., page 447.*)

x, p 10.

**Methods of Diagnosis.**—F. W. Russell[1] draws attention to some points in estimating the motor power of the stomach. When a test meal of a pint of fluid is given, if more than two ounces can be withdrawn by the stomach tube an hour afterwards, that fact alone is some evidence of dilatation. Again, if the salol test is given two hours after food salicylates appear in the urine in health at from forty to seventy-five minutes later and should disappear in thirty hours, but if the stomach contents do not remain acid the reaction may appear much sooner, even in fifteen minutes, provided the bladder be emptied of previous urine. A meal of alkaline food soon after the test may shorten the duration of the reaction; and, on the other hand, he finds that elimination sometimes continues more than thirty hours without any other evidence of gastric disorder. A. L. Benedict[2] has also found salicylates in the urine forty-eight hours after a dose of salol, though the motor power seemed good from the appearance of salicylates as early as thirty minutes after a dose. He regards the stomach tube as chiefly useful in determining the amount of H Cl and in the treatment of dilatation. J. L. Salinger[3] treated sixty-seven patients with various gastric disorders by lavage through the tube alone, and found fifty-two much improved. Even in cancer great alleviation was obtained. Déleage[4] points out that lavage is dangerous in ulcer, varices, and very friable cancer of the œsophagus, hemiplegia, arterio-sclerosis, and advanced heart disease. However, its value is very great in proper cases, such as those of dilatation. Thus, J. Michell Clarke[5] has found that a single washingout in mild attacks and daily treatment with warm boracic solution before breakfast in others is most important for effecting a cure. He insists on careful diet, at first frequent small doses of peptonised milk, and finally three meals only in the day of easily digested food, such as leaves a small residue behind. Very little fluid is given by him with these meals, but in many cases a tumbler of hot water before breakfast is useful to promote the contractility of the stomach. A mixture containing strychnia, H Cl, and chloroform water, or bismuth and salicylate of soda, may be administered during the treatment. In diagnosing dilatation or cancer, the motor power is, he thinks, best estimated by introducing the tube at the time when the stage of digestion should be normally completed, so as to estimate the residue. On the whole, he confirms the views mentioned above as to the presence of lactic acid, and the absence of H Cl in most cases of cancer, and employs Uffelman's reagent for the former acid, and Gunsberg's for H Cl as fairly reliable tests. Moritz[6] proves that pure water is passed on very rapidly from the stomach to the intestine, but that äerated water and all other liquids much more slowly. Soups provoke a strongly acid reaction, and are passed on very slowly. Little, if any, absorption of pure water takes place from the stomach. A number of cases of dilatation treated by washing out the stomach with rapid improvement are reported by E. Cureton[7] from the Salop Infirmary. The solutions used were plain boiled water, or those made with bicarbonate or sulpho-carbonate of soda or sulphurous acid. The relief from pain and vomiting was marked, and the patients quickly gained weight, and many of them learned to pass the tube themselves. Careful diet and gastric tonics were given in addition. In acid dyspepsia the saliva produced after mastication has ceased tends to become acid, but J. Bergmann[8] has shown that if tablets of some simple substance be slowly chewed for a considerable time a profuse flow of alkaline saliva is formed, sufficient to neutralise the superacidity of the stomach, and this treatment is found sufficient in some cases of this disorder. This, too, probably explains in part the efficacy of the bismuth lozenge, which Sir William Roberts recommends to be sucked slowly between meals. Where more elaborate methods of diagnosis are impracticable, much can be learnt from the sensations experienced after a test breakfast of a pint of weak tea with milk and a little sugar and a roll of bread. If two hours later there is a sense of heat, burning, and acidity, we have probably to do with hyperacid dyspepsia. The burning is often felt at night between eleven and one a.m., and often the ingestion of food gives temporary relief. If on the other hand the meal is followed by a sense of weight and abundance of gas, there is hypoacid dyspepsia. If after a quarter of an hour there is epigastric pain gradually increasing, there is probably gastro-duodenal irritation. Finally cramps, pain, and vomiting may occur, pointing to muscular and nervous disorders.[9] Gastric atrophy is occasionally met with, either from carcinoma or as a primary affection forming the last stage of chronic gastritis. The symptoms, according to Schmidt,[10] are extreme anæmia, failure of secretory and sometimes of the motor powers of the stomach, and attacks of severe pain with vomiting.

**Minor Affections.**—The successful treatment of an ordinary "bilious attack," providing it is not a mere symptom of more serious troubles such as uræmia, requires a careful diet for a few days. We should, says Saundby,[11] exclude especially starch foods, fat, and sugar, as well as pepper, spirits, undiluted wine, vegetables, and brown bread. For breakfast we may give tea, cocoa, or coffee with plenty of milk, a little toast, and broiled fish; for luncheon white fish, chicken or mutton, toast, and custard pudding or blancmange; and a similar list for dinner, avoiding butter, vegetables, and soups. A blue pill and a tumbler of hot water before breakfast with or without Carlsbad salts, and a powder of soda, bismuth, and cinnamon before meals will be found efficacious. In severe attacks it may be needful to insist on a diet of milk and lime water for a day or two. The true pathology is not the sluggish liver of popular parlance, but a catarrhal inflammation of the stomach from irritating food and drink. Stephen Mackenzie[12] regards ordinary flatulence as due not to fermentation, but to want of tonicity of the stomach from impaired nervous vigour, often associated with gastric catarrh. Strychnia or nux vomica with general treatment, and a pill containing carbolic acid are indicated. For violent attacks of "spasms" he recommends Sp.

cajuputi, Sp. ammon. aromat., Sp. chloroform aa m xx. To be given in a wineglassful of water every half-hour until relief is obtained. The difficulty of stopping vomiting in nervous subjects and after operations has been sometimes met by the use of acetanilide, two grains every hour for three doses,[13] and the effects are described as very marked. The use of chlorobrom in sea sickness has been highly praised by several writers, but a contributor in the *Lancet* insists that it is too depressing for severe cases, and would rely instead on minute doses of morphia.

Gautier and Larat[14] report that the descending continuous current is of great value in stopping intractable nervous vomiting. The application should be continued from fifteen to forty-five minutes several times a day. The vomiting of pregnancy is not relieved by it, but otherwise, except there is an organic lesion in the stomach, the results are uniformly good.[15] Two fatal cases of Anorexia nervosa have been recently described. As usual, they occurred in girls who persisted in refusing food, became extremely emaciated, with slow pulse and respiration, subnormal temperature, and much restlessness. Neither diabetes, phthisis, or other complications could be detected, and the strength was well maintained till just before the end.[16] Occasionally, in healthy persons we meet with uncontrollable vomiting after meals, which may be preceded by paroxysms of coughing. J. Dunn rightly draws attention to the fact that many of these cases are due to elongated uvula, or an inflamed papilla at the base of the tongue. Another rare condition is that of croupous gastritis, where a membranous cast of the interior of the stomach may be vomited. J. Thomson reports an instance in a weakly child, and thinks it usually occurs in patients whose vitality is exhausted by severe disease.[18]

[1] Birming. Med. Rev., Feb., 1895. [2] Ther. Gaz., p. 587, 195. [3] Ibidem, p. 592. [4] Med. Week, May 10. [5] Bristol Med. J., Sept. [6] Birming. Med. Rev., June. [7] Lanc., Aug. 24. [8] Berlin Klein. Woch.. 32, 95. [9] Med. Rec., Nov. 24, 1894. [10] B. M. J., June 29, 1895. [11] Clin. J., Ap. 17. [12] Practit., July. [13] Med. Rev., Feb. 9. [14] Lanc., Jany. 19. [15] Med. Week, Ap. 19. [16] Lanc., Jany. 5 and 19. [17] N. Y. Med. J., Sept. 22, 1894. [18] Interl. Med. Mag., May, 1895.

